# Giant Aortic Root Thrombus in a Chronic, Asymptomatic Stanford Type A Aortic Dissection

**DOI:** 10.1016/j.jaccas.2024.102249

**Published:** 2024-04-17

**Authors:** Laura Baquero, Hilda Loaisiga, Eduardo Alvarado

**Affiliations:** a Cardiology Fellowship, University of Costa Rica, San Jose, Costa Rica; b Department of Cardiology, Hospital San Vicente de Paul, San Jose, Costa Rica

**Keywords:** aorta, aortic valve, dissection

## Abstract

Type A aortic dissection rarely becomes chronic because of high early mortality. Thrombus in the false lumen and an immobile flap are indicative of this condition. A 61-year-old man with an initial diagnosis of gastroenteritis later presented with a diastolic murmur. Echocardiography revealed chronic Stanford A aortic dissection with a thrombus causing severe aortic regurgitation.

## Graphical abstract

**Figure:**
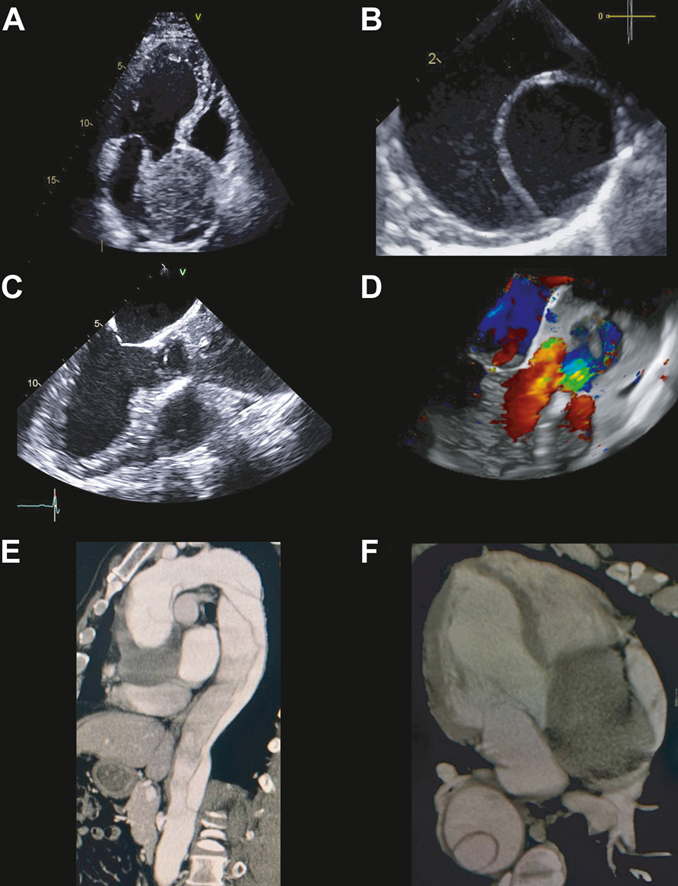

A 61-year-old man presented to the emergency department with a report of an acute onset of chest pain described as stabbing discomfort radiating to the back and epigastric region. A provisional diagnosis of acute gastroenteritis was initially made, after which he was discharged. Over the next 2 years, the patient remained asymptomatic. Results of a comprehensive blood count, baseline metabolic profile, and electrocardiogram were all within normal limits.

A diastolic aortic murmur discovered during a routine follow-up visit prompted an echocardiographic study. The transthoracic echocardiogram showed not only preserved left ventricular function with an ejection fraction of 57% but also an ascending thoracic aortic aneurysm measuring 6.6 cm. A substantial thrombotic mass occluded almost the entire aortic lumen (Figure 1A, Supplemental Figures 1 to 3). These findings were confirmed by transesophageal echocardiography (Figures 1B to 1D). The dissection was found to have started at the level of the aortic sinus, characterized by an echogenic mass corresponding to an extensive thrombus within the false lumen and causing severe aortic regurgitation without the presence of significant stenosis.

**Figure 1:**
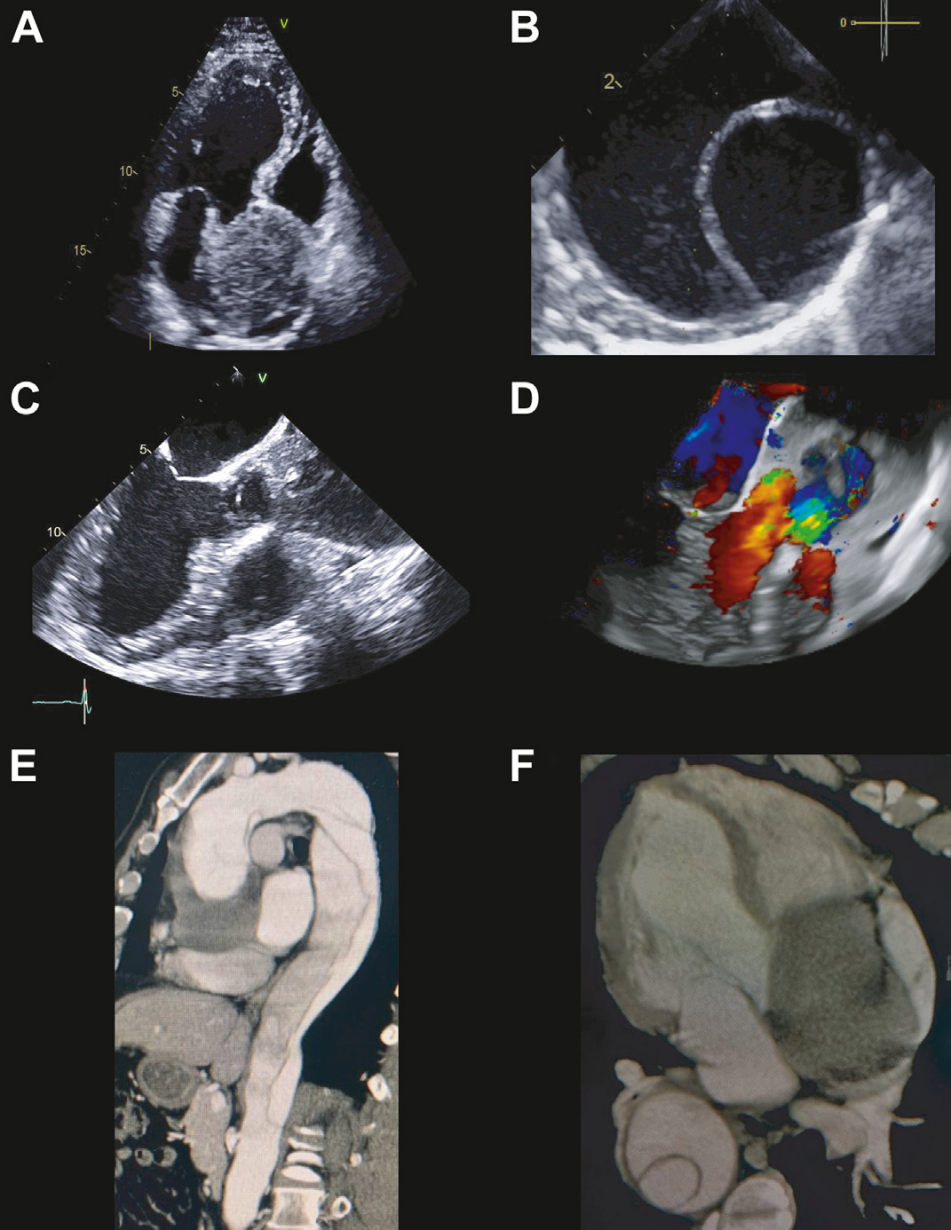
Multimodality Imaging of Massive Thrombus in Chronic DeBakey I, Stanford Type A Aortic Dissection (A) Presurgery transthoracic echocardiogram, in the apical long-axis view, shows the thrombus in the aortic root. (B) Transesophageal echocardiogram demonstrates the thickness of the dissection flap in the thoracic aorta, thus denoting chronicity. (C and D) Transesophageal echocardiogram in 2 and 3 dimensions confirms the documented findings, and it also reveals an ascending thoracic aortic aneurysm measuring 6.6 cm and severe aortic regurgitation that is caused by a mixed mechanism: aortic dilatation and thrombus occupation of the aortic root. (E and F) Computed tomography reaffirms the findings and establishes the extent of the dissection.

Subsequent computed tomography confirmed the diagnosis of chronic fusiform DeBakey type I, Stanford type A aortic dissection (AD) (Supplemental Figures 4 and 5). It delineated a semilunar mural thrombus measuring 2.6 × 6 × 6.1 cm, with the dissection flap extending from the aortic root to the external iliac arteries (Figures 1E and 1F).

Following the 2020 reporting standards defined by the Society for Vascular Surgery and The Society of Thoracic Surgeons, AD chronicity is classified into hyperacute (<24 hours), acute (1-14 days), subacute (15-90 days), and chronic (>90 days) phases.^1^ Chronic AD is divided into 3 groups: 1) patients who have survived the acute phase of type AD and have undergone surgical intervention but have retained a false lumen; 2) patients who have undergone complete recovery after surgery; and 3) an unusual subset of patients who progress from an undiagnosed acute phase to a chronic state.^2^

This last group, consisting of patients who have survived the acute phase of type A AD, experiences a transition to a chronic state. In this phase, the disease can either follow a stable clinical course or develop with complications such as progressive degeneration, malperfusion, or severe aortic regurgitation, as in the present case. These patients may either remain asymptomatic or experience symptoms secondary to resulting complications, including visual disturbances or syncope caused by malperfusion syndrome or dyspnea from aortic insufficiency. In patients with complications, surgery is recommended, whereas in patients with stable disease, wait-and-see management may be considered.^2^ In accordance with the American Heart Association and American College of Cardiology guidelines for aortic disease, elective thoracic aortic repair is recommended in patients with a history of acute AD treated conservatively when the aneurysm diameter reaches or exceeds 5.5 cm.^1^ In addition, cardiac imaging is an important diagnostic tool, particularly in the presence of thrombus in the false lumen, along with a thickened, immobile dissecting flap, which is a clear sign of chronicity, as in the present case.^3^

Subsequently, the patient underwent successful Bental de Bono surgery, which resulted in favorable postoperative convalescence. This case serves as an example of an atypical clinical course in which diagnostic imaging played a critical role in identifying chronic type A AD associated with a substantial thrombotic mass and severe aortic regurgitation, ultimately leading to successful surgical intervention.

## Funding Support and Author Disclosures

The authors have reported that they have no relationships relevant to the contents of this paper to disclose.
