# Supplementary material for: Giant Aortic Root Thrombus in a Chronic, Asymptomatic Stanford Type A Aortic Dissection
Source: JACC Case Rep. 2024 Apr 17;29(8):102249. doi: 10.1016/j.jaccas.2024.102249 (PMC11103594; doi:10.1016/j.jaccas.2024.102249)
Supplement: Supplemental Figures 1-5 [file mmc1.docx]

**
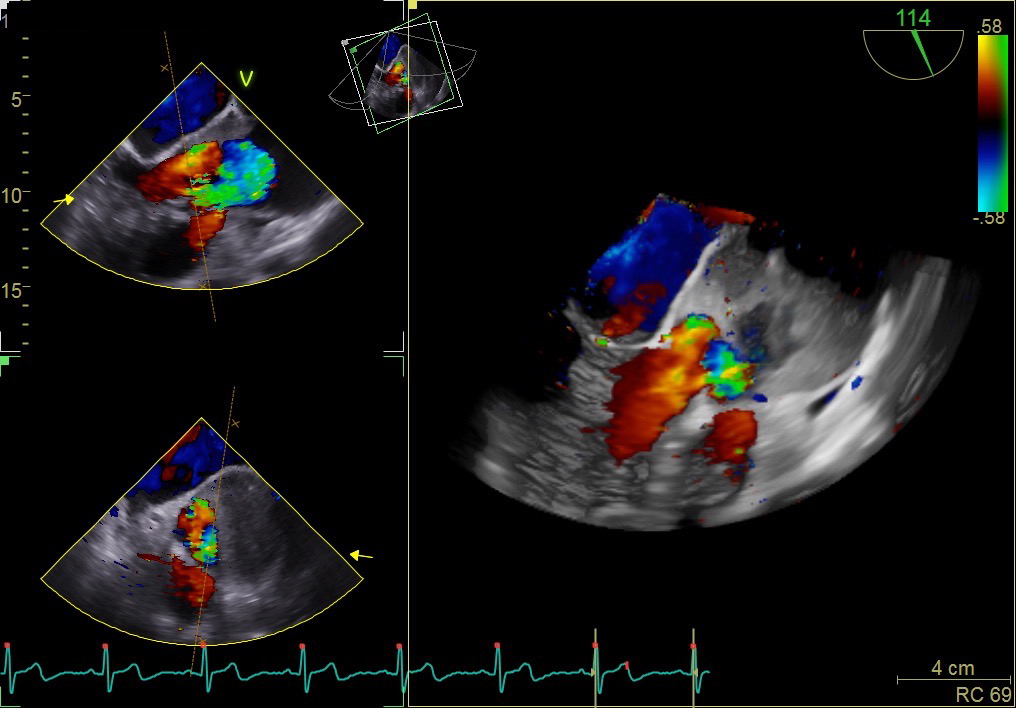
**

**Supplemental Figure 1:** Aortic Regurgitation on transesophageal echocardiogram. Transesophageal echocardiogram in 2D and 3D establishes the presence of severe aortic regurgitation caused by the aortic dilatation and the chronic thrombus in the false lumen.


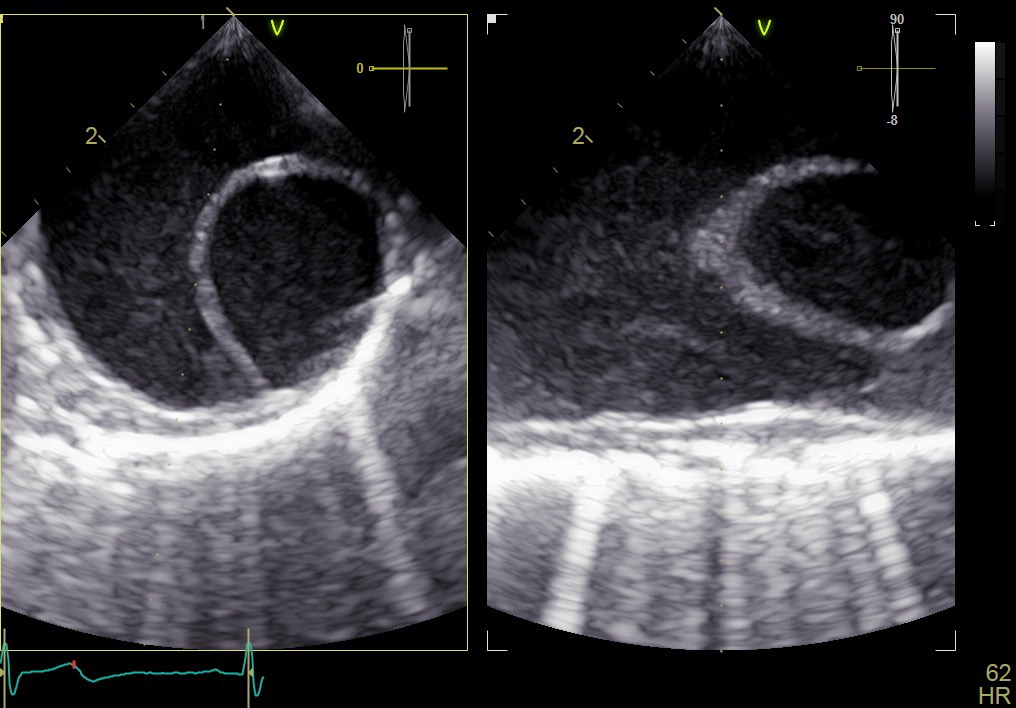


**Supplemental Figure 2:** Chronic Aortic Dissection on Descending Aorta. Transesophageal echocardiogram denotes the characteristics of a chronic aortic dissection.


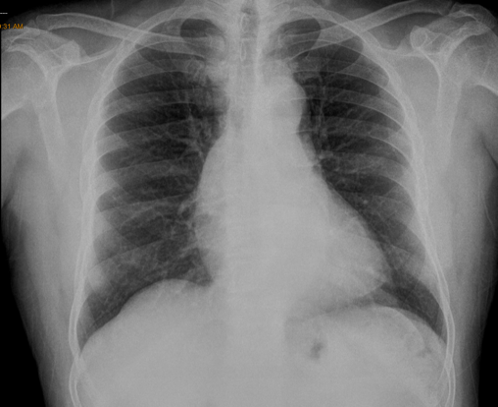


**Supplemental Figure 3.** Aortic Aneurysm on Chest Radiography. Posteroanterior chest radiography shows a prominent mediastinum caused by the aortic aneurysm and dissection.


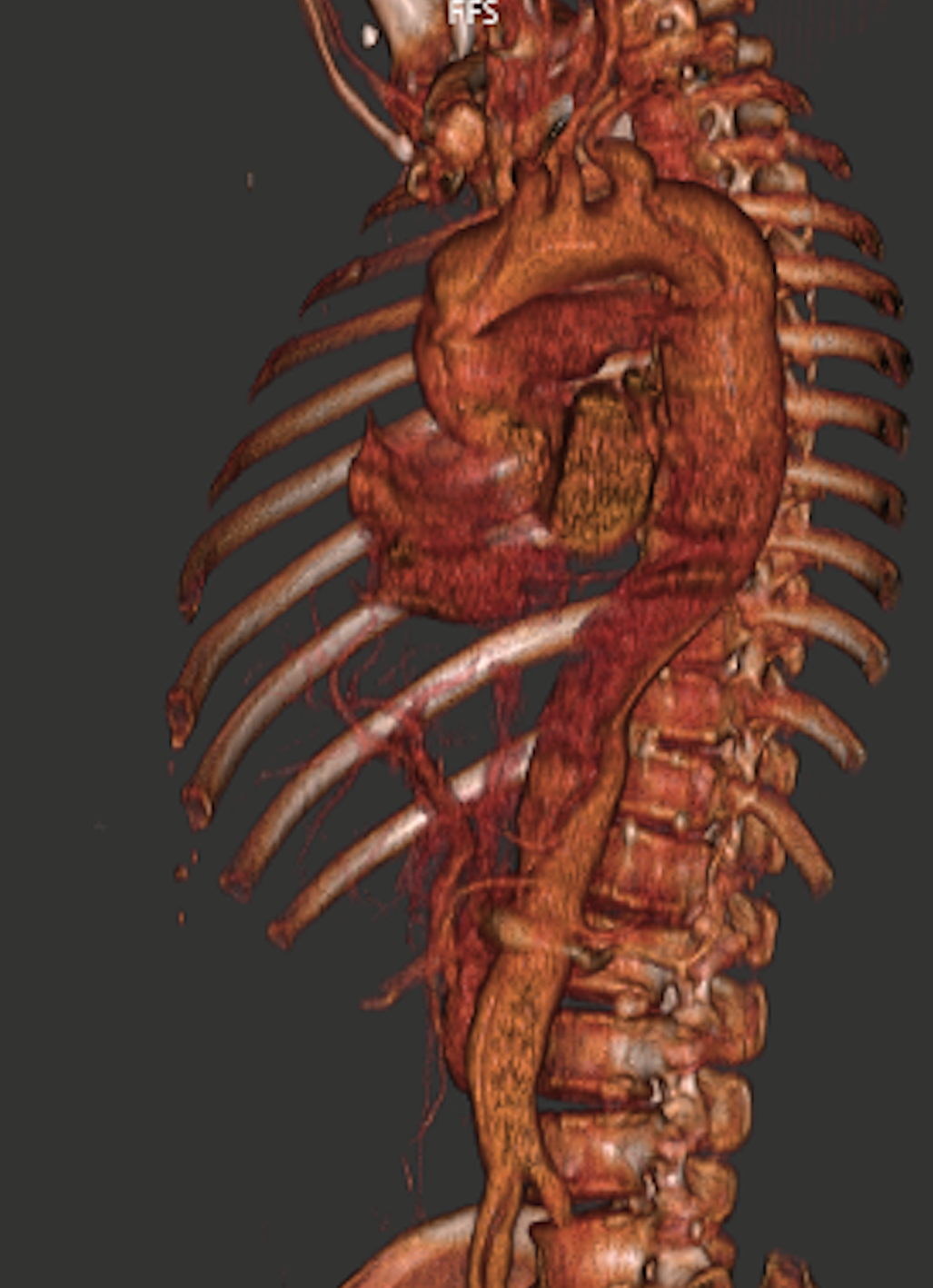


**Supplemental Figure 4.** 3D CT reconstruction of chronic aortic dissection. 3D CT reconstruction of chronic Stanford A, DeBakey I aortic dissection.


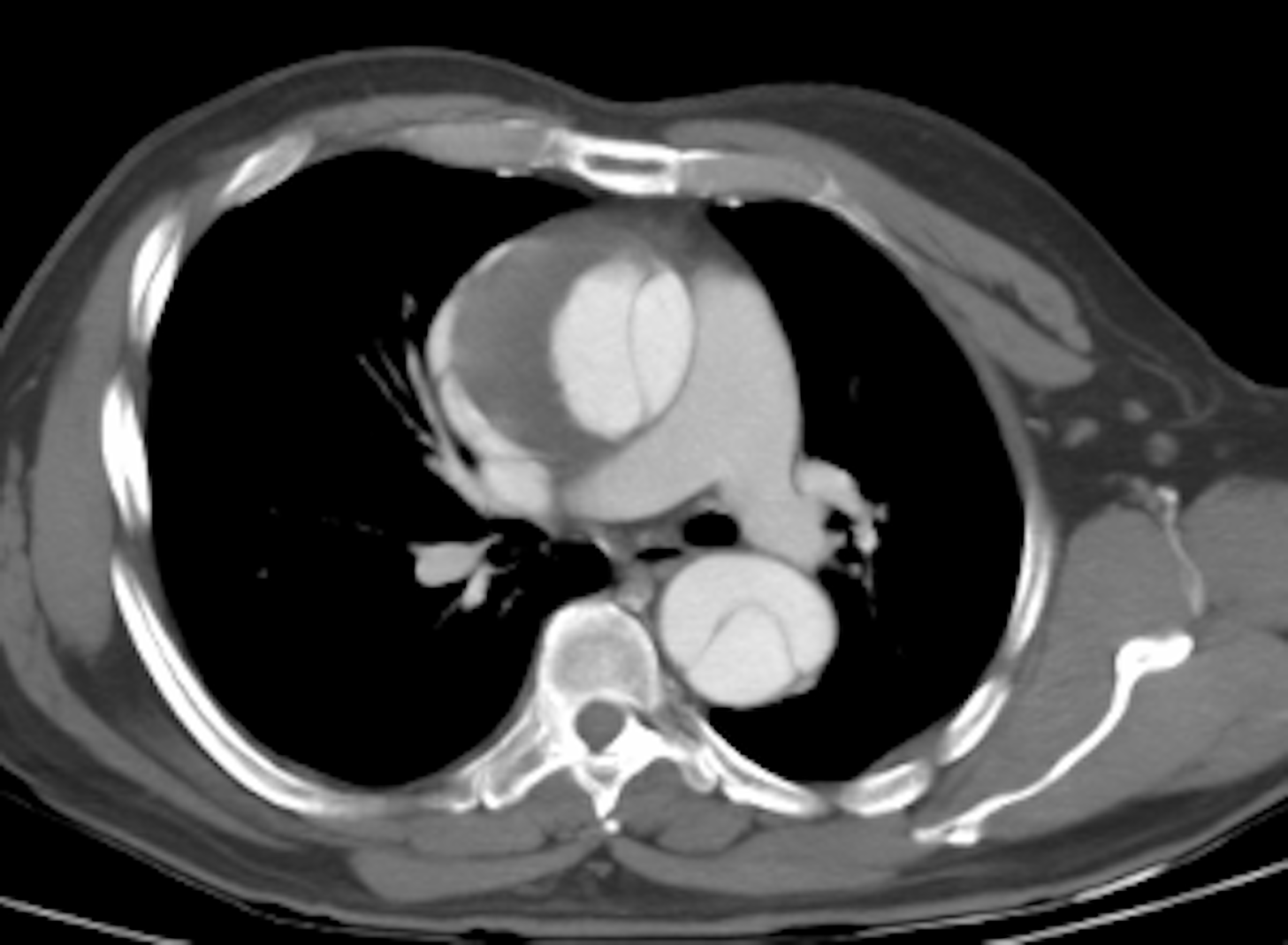


**Supplemental Figure 5.** Chronic aortic dissection on thoracic angiotomography.
